# Performance of a capnodynamic method estimating cardiac output during respiratory failure - before and after lung recruitment

**DOI:** 10.1007/s10877-019-00421-w

**Published:** 2019-11-20

**Authors:** Thorir Svavar Sigmundsson, Tomas Öhman, Magnus Hallbäck, Eider Redondo, Fernando Suarez Sipmann, Mats Wallin, Anders Oldner, Caroline Hällsjö-Sander, Håkan Björne

**Affiliations:** 1 Function Perioperative Medicine and Intensive Care, Karolinska University Hospital, 171 76 Solna, Stockholm Sweden; 2 Department of Physiology and Pharmacology, Karolinska Institutet, Stockholm, Sweden; 3 Maquet Critical Care AB, Solna, Sweden; 4 Department of Intensive Care Medicine, Complejo Hospitalario de Navarra, Pamplona, Spain; 5 Department of Surgical Sciences, Section of Anaesthesiology and Critical Care, Hedenstierna’s Laboratory, Uppsala University, Uppsala, Sweden; 6 CIBER de Enfermedades Respiratorias, Instituto de Salud Carlos III, Madrid, Spain

**Keywords:** Carbon dioxide, Cardiac output, Effective pulmonary blood flow, Capnodynamic, Lung injury, Respiratory failure, Animal model

## Abstract

Respiratory failure may cause hemodynamic instability with strain on the right ventricle. The capnodynamic method continuously calculates cardiac output (CO) based on effective pulmonary blood flow (CO_EPBF_) and could provide CO monitoring complementary to mechanical ventilation during surgery and intensive care. The aim of the current study was to evaluate the ability of a revised capnodynamic method, based on short expiratory holds (CO_EPBFexp_), to estimate CO during acute respiratory failure (LI) with high shunt fractions before and after compliance-based lung recruitment. Ten pigs were submitted to lung lavage and subsequent ventilator-induced lung injury. CO_EPBFexp_, without any shunt correction, was compared to a reference method for CO, an ultrasonic flow probe placed around the pulmonary artery trunk (CO_TS_) at (1) baseline in healthy lungs with PEEP 5 cmH_2_O (HL_P5_), (2) LI with PEEP 5 cmH_2_O (LI_P5_) and (3) LI after lung recruitment and PEEP adjustment (LI_Padj_). CO changes were enforced during LI_P5_ and LI_Padj_ to estimate trending. LI resulted in changes in shunt fraction from 0.1 (0.03) to 0.36 (0.1) and restored to 0.09 (0.04) after recruitment manoeuvre. Bias (levels of agreement) and percentage error between CO_EPBFexp_ and CO_TS_ changed from 0.5 (− 0.5 to 1.5) L/min and 30% at HL_P5_ to − 0.6 (− 2.3 to 1.1) L/min and 39% during LI_P5_ and finally 1.1 (− 0.3 to 2.5) L/min and 38% at LI_Padj_. Concordance during CO changes improved from 87 to 100% after lung recruitment and PEEP adjustment. CO_EPBFexp_ could possibly be used for continuous CO monitoring and trending in hemodynamically unstable patients with increased shunt and after recruitment manoeuvre.

**Electronic supplementary material:**

The online version of this article (10.1007/s10877-019-00421-w) contains supplementary material, which is available to authorized users.

## Introduction

Hemodynamically unstable patients with severe respiratory failure constitute a medical challenge. In these patients, the interaction between the heart and lungs is frequently affected with increased pulmonary vascular resistance (PVR) and potential right ventricular failure [1]. Lung protective mechanical ventilation commonly improves compliance and oxygenation. However, the accompanying high levels of PEEP can increase the right ventricular afterload with negative effect on the cardiac performance [2, 3]. Hemodynamic monitoring is therefore of great importance to optimize blood perfusion to the injured lungs with least possible strain on the right ventricle.

The capnodynamic method (CO_EPBF_) continuously calculates pulmonary blood flow (CO minus the shunted blood fraction) utilizing small variations in partial pressure of alveolar CO_2_ (P_A_CO_2_), automatically controlled by the ventilator [4, 5]. As previously described, the capnodynamic method with inspiratory holds (CO_EPBFinsp_) showed acceptable performance in animals with low shunt [4], however when the animals were subjected to lavage-induced lung injury both accuracy and precision were affected [6]. After refinement of the method with the obliged holds inserted in expiration (CO_EPBFexp_) instead of inspiration the performance in a porcine model with healthy lungs improved with sustained good trending ability during hemodynamic and ventilatory challenges [5, 7–9]. In addition, CO_EPBFexp_ has been shown to provide stable CO monitoring in healthy infants undergoing cleft-lip surgery [8].

Complementary monitoring of pulmonary blood flow during mechanical ventilation in respiratory failure could assist clinicians to perform lung recruitment and adjust PEEP levels, with regards to the right ventricular heart function. [5, 10]. The aim of this study was to evaluate the modified capnodynamic method based on expiratory holds at increased shunt levels during lung injury, before and after lung recruitment and under major hemodynamic changes.

## Methods

The study was approved by the Uppsala animal research ethical committee (nr. C 47/15) and performed at the Hedenstierna laboratory in Uppsala University, Sweden. The animals used in this study were collected from the same breeding colony (Mångsbo Farm, Uppsala, Sweden). At the farm they had unlimited access to tap water and food on a standardized schedule and kept in a light and temperature-controlled environment. Eight of them were included in another study protocol analysing the effects of ischemia and reperfusion [7]. Results are reported according to the GRRAS guidelines [11].

### Anaesthesia and preparation

Briefly, 10 pigs with a mean weight of 38 kg (range 35–44 kg) were anaesthetised and mechanically ventilated in a volume-controlled mode with a tidal volume (TV) of 8 mL/kg, FiO_2_ 0.40 and PEEP 5 cmH_2_O (Servo-i, Maquet Critical Care, Solna, Sweden). At baseline respiratory rate (RR) was adjusted to normal ventilation according to blood gas analysis. An arterial catheter and a pulmonary artery catheter (Edwards Lifesciences Corp., Irvine, CA, USA) were inserted with a cut down in the neck under direct vision. An inflatable thrombectomy catheter (Dispomedica GmbH, Hamburg, Germany) and a stent graft balloon catheter (Reliant®, Medtronic Inc. Minneapolis, MN, USA) were inserted with ultrasound guidance in the inferior caval vein for controlled preload reduction and the caudal aorta (not used in this protocol), respectively. An ultrasonic flow probe (AUseries Confidence Flowprobe^®^ with ultrafit liner, Transonic Systems Inc., Ithaca NY, USA) to measure CO (CO_TS_) was surgically inserted around the pulmonary trunk (CO_TS_) through a left sided thoracotomy.

A mainstream infrared sensor (Capnostat-3, Respironics Inc, Wallingford, CT, USA) was used to measure concentration of expired CO_2_. Gas flow was analysed by the flow sensor incorporated in the ventilator and transmitted to a computer where all the mathematical analysis was carried out with a software written in Matlab™ (The Mathworks Inc, Natick, MA, USA).

ABL-800FLEX (Radiometer Medical ApS, Brønshøj, Denmark) was used for blood gas analyses. Hemodynamic parameters were retrieved into a data acquisition system (Acknowledge, version 3.2.7, Bio Pac Systems, Santa Barbara, CA, USA). Core temperature was maintained at 38–39 °C. Animals were sacrificed with potassium chloride injection at the end of the protocol.

### Calculations and measurements of cardiac output, shunt and dead space

A detailed description of the capnodynamic equation can be found in the supplementary material. As previously described [5, 7], a short pause is introduced to the expiratory phase of three out of nine breaths, automatically controlled by the ventilator. The resulting small differences (0.5–1 kPa) in the alveolar concentration of CO_2_ between breaths can be inserted into the capnodynamic equation, describing the mole balance of CO_2_ transported to and from the lungs. Each breath creates one equation and with a stack of nine equations the CO_EPBF_ can be calculated using a least square-error optimization. With each breath the last equation is replaced with the newest allowing a continuous calculation of CO_EPBF_ with each presented value representing an average of the preceding nine breaths (approximately 20 s).

The experimental reference method, CO_TS_, represents the flow generated by each cardiac cycle measured at the pulmonary trunk and is considered the gold standard for invasive CO measurement. Each CO reading was performed during a steady state were CO_EPBF_ represents the CO for the preceding 9 breaths (~ 20 s) and COTS the preceding 5–10 s.

The pulmonary artery catheter (PAC) was used for mixed venous blood sampling, gas analysis and calculation of hemodynamic parameters. Blood samples were drawn after the CO_TS_ and CO_EPBF_ readings to avoid disturbances on the CO_2_ signal.

Shunt fraction was calculated using Berggren’s formula [12]. Physiological dead space (Vd/Vt), ad modum Enghoff, representing the global V/Q mismatch in the lungs was measured with PaCO_2_ and volumetric capnography (NICO monitor, Respironics, Wallingford CT, USA) [13].

### Lung injury

Ten animals were subjected to a two-hit ventilation induced lung injury (VILI) model, approximately 2 h after severe ischemia and reperfusion, as previously reported [7]. Repeated lung lavages with 37° isotonic saline (30 ml/kg) were combined with subsequent 30–60 min of injurious mechanical ventilation combining zero PEEP with an inspiratory pressure of 30–35 cmH_2_O.

#### Experimental protocol

During the protocol, TV and RR were left unchanged. CO_EPBF_ and CO_TS_ data was collected continuously breath by breath (CO_EPBF_) and beat to beat (CO_TS_). Data readings for CO_EPBF_ and CO_TS_ were registered simultaneously in the case report file during steady states. The first reading was registered at PEEP 5 (HL_P5_) at the start of the day and subsequently after lung injury (LI_P5__BL 1), controlled preload reduction with caval balloon inflation (LI_P5__CAVA), Dobutamine infusion (LI_P5__DOB), aiming for ± 30% change in CO. Baseline measurements were performed before and after changes in CO (LI_P5__BL 2 and 3). Thereafter a recruitment manoeuvre (RM) was performed where the level of PEEP resulting in maximum dynamic compliance was considered the closing pressure. PEEP adjusted was set at 2–3 cmH_2_O higher than the closing pressure. The lung recruitment procedure lasted for 22 ± 14 min and resulted in a PEEP range of 11–17 cmH_2_O. CO_EPBF_ and CO_TS_ readings were repeated after PEEP adjustment during steady state at baseline (LI_Padj_ BL 4), caval balloon inflation (LI_Padj__CAVA), baseline (LI_Padj__BL 5) again and Dobutamine infusion (LI_Padj__DOB) as described before. Fluid and vasopressor treatment were adjusted to maintain stability and time between each intervention was 7–15 min depending on time to stabilisation.

### Statistics

Data was analysed for normal distribution with D’Agostino and Pearson omnibus K2 test and proportional bias, i.e. the spread of bias at different CO levels, was checked with visual assessment and by a linear regression. Results are presented as mean (standard deviation, SD). A *p* value of < 0.05 was considered significant. Statistical calculations were performed in Graph Pad Prism (version 6.0 for Windows, Graph Pad Software, La Jolla, CA, USA). Cartesian data for polar plots was converted to polar coordinates in an Excel sheet (kindly provided by Professor L. Critchley) and displayed as graphs in Medcalc Statistical Software version 16.8.4 (MedCalc Software bvba, Ostend, Belgium) [14]. Calculations of all confidence intervals (CI) were performed in Excel (version 2007).

Correction for repeated measurements was not applied as each measurement was considered independent with time for stabilization during and between each hemodynamic intervention [15, 16].

### Precision

Individual precision (defined as twice the coefficient of variation (CV = SD_method_/mean CO_method_) of CO_EPBFexp_ and CO_TS_ was calculated at baseline conditions using ten measurements obtained at 1-min intervals in each animal [17]. Our previously reported precision for CO_EPBFexp_ was 8 to 14% during steady state conditions and 4% for the CO_TS_ [5, 7].

### Absolute values and percentage error

Bland–Altman methodology was used to measure the mean difference (bias) between the methods and the precision (levels of agreement) [18–20]. Percentage error (PE) to estimate the accuracy was calculated as $100\% \times 1.96 \times \frac{\text{SD}}{{{\text{mean}} {\text{CO}}}}$ [15, 20], where SD is the standard deviation of the difference between the methods and mean CO is the mean cardiac output of the reference method.

A priori, CO_EPBF_ was considered interchangeable to CO_TS_ if percentage error was < 30% [20].

### Trending ability

The agreement in the direction and magnitude of the change was assessed with a four-quadrant and polar plot methodologies by dividing the number of data points within the two quadrants of agreement and the radial limits of agreement of ± 30° with the total number of data points [14]. Because of the high precision of the reference method, an exclusion zone of 10% was used [21]. Concordance rates of > 92% and > 90% calculated by the four-quadrant plot and the polar plot respectively, were considered good [22]. An angular bias smaller than ± 5° indicated good calibration between the test and the reference method [14, 22].

## Results

Data from two animals were excluded in the analysis; one animal was critically unstable after the lung injury and measurements before lung recruitment were not possible. In the second animal a computer failure made CO_EPBF_ calculation impossible.

Lung injury resulted in respiratory failure including decreased dynamic compliance and ratio of partial pressure of oxygen in arterial blood to inspired fraction of oxygen (P_a_O_2_/F_i_O_2_), as well as increased shunt, physiological dead space and partial pressure of CO_2_ in mixed venous blood (P_v_CO_2_). The individual recruitment manoeuvre with PEEP adjustment to 15 (3) cmH_2_O (range 11–17 cmH_2_O), normalized shunt and compliance to large extent, although physiological dead space and P_v_CO_2_ were elevated compared to baseline (see Table 1 for changes in respiratory and hemodynamic parameters during the protocol).

**Table 1:** Hemodynamic parameters at different conditions during the protocol

	PEEP 5	LI PEEP 5					RM	LI PEEP adj			
	HL	BL 1	CAVA	BL 2	DOB	BL 3		BLPadj	CAVA	BLPadj	DOB
CO_TS_ (L/min)	3.5 (0.5)	4.4 (0.6)	2.9 (0.5)	4.5 (0.7)	5.7 (0.8)	4.3 (0.8)		3.6 (0.4)	2.5 (0.3)	3.6 (0.4)	4.7 (0.4)
CO_EPBF_ (L/min)	4.0 (0.5)	3.5 (0.7)	2.6 (0.5)	4.0 (0.7)	4.6 (0.7)	4.1 (0.4)		4.7 (0.6)	3.5 (0.5)	4.8 (0.5)	5.8 (0.6)
HR (beats/min)	84 (8)	114 (6)	124 (4)	115 (4)	133 (9)	118 (8)		117 (7)	124 (12)	116 (10)	133 (13)
MAP (mmHg)	74 (10)	73 (9)	57 (9)	83 (15)	78 (11)	75 (11)		83 (16)	60 (11)	90 (18)	89 (8)
mPAP (mmHg)	19 (1)	28 (3)	23 (2)	29 (3)	30 (3)	30 (3)		26 (2)	22 (3)	27 (3)	28 (3)
SVR (dynes/s/cm^−5^)	1525 (435)	1180 (279)	1425 (322)	1362 (481)	974 (232)	1242 (429)		1610 (471)	1531 (258)	1768 (522)	1331 (225)
PVR (dynes/s/cm^−5^)	154 (66)	252 (54)	394 (134)	286 (130)	217 (97)	284 (101)		231 (57)	265 (44)	238 (58)	193 (60)
Shunt (fraction)	0.1 (0.03)	0.36 (0.1)	0.26 (0.1)	0.38 (0.07)	0.41 (0.07)	0.33 (0.08)		0.09 (0.04)	0.09 (0.04)	0.09 (0.02)	0.14 (0.06)
Vd/Vt (fraction)	0.55 (0.03)	0.75 (0.05)	0.76 (0.05)	0.72 (0.04)	0.74 (0.04)	0.68 (0.05)		0.63 (0.05)	0.67 (0.06)	0.64 (0.06)	0.66 (0.06)
PvCO_2_ (kPa)	7.2 (0.7)	9.4 (1.2)	9.6 (1.3)	9.5 (1.5)	9.7 (1.4)	9.1 (0.7)		8.7 (0.9)	9.0 (1.6)	8.3 (0.8)	8.7 (1.6)
EtCO_2_ (kPa)	5.8 (0.7)	5.5 (0.3)	5.0 (0.5)	5.5 (0.4)	5.9 (0.4)	5.7 (0.2)		6.1 (0.7)	5.5 (0.7)	6.0 (0.6)	6.2 (0.7)
PaO_2_/FiO_2_ (kPa)	391 (54)	121 (68)	149 (92)	141 (75)	139 (50)	184 (83)		419 (180)	472 (86)	441 (187)	460 (103)
V_T_ (ml)	312 (17)	310 (12)	310 (12)	310 (12)	310 (12)	309 (11)		310 (12)	310 (12)	310 (12)	310 (13)
RR (1/min)	31 (2)	31 (3)	31 (3)	31 (3)	31 (3)	31 (3)		32 (3)	32 (3)	32 (3)	32 (3)
Cdyn (ml/cmH_2_O)	36 (6)	17 (2)	17 (2)	18 (2)	18(2)	17 (2)		33 (4)	32 (5)	32 (4)	31 (5)
PEEP (cmH_2_O)	5 (1)	6 (1)	6 (1)	5 (1)	6 (1)	5 (1)		14 (3)	14 (3)	14 (3)	14 (3)
DP (cmH_2_O)	13 (1)	20 (1)	19 (2)	19 (2)	19 (2)	19 (2)		15 (2)	15 (2)	15 (2)	15 (2)
ELV (ml)	875 (165)	531 (141)	575 (81)	565 (105)	617 (87)	592 (101)		948 (325)	923 (294)	923 (309)	1000 (339)

*HL* healthy lungs, *BL* baseline, *LI* lung injury, *CAVA* preload reduction with balloon inflation in the vena cava, *Dob* dobutamine infusion, *HR* heart rate, *MAP* mean arterial pressure, *mPAP* mean pulmonary artery pressure, *SVR* systemic vascular resistance, *PVR* pulmonary vascular resistance, *Vd/Vt* physiological dead space (Enghoff modification), *PvCO*_2_ partial pressure of CO_2_ in mixed venous blood, *EtCO*_*2*_ end tidal CO_2_, *PaO*_*2*_*/FiO*_*2*_ ratio of arterial oxygen partial pressure to fractional inspired oxygen, *VT* tidal volume, *RR* respiratory rate, *Cdyn* dynamic compliance, *DP* driving pressure, *ELV* effective lung volume (CO_2_-based lung volume calculated by the capnodynamic method)

**Fig. 1:**
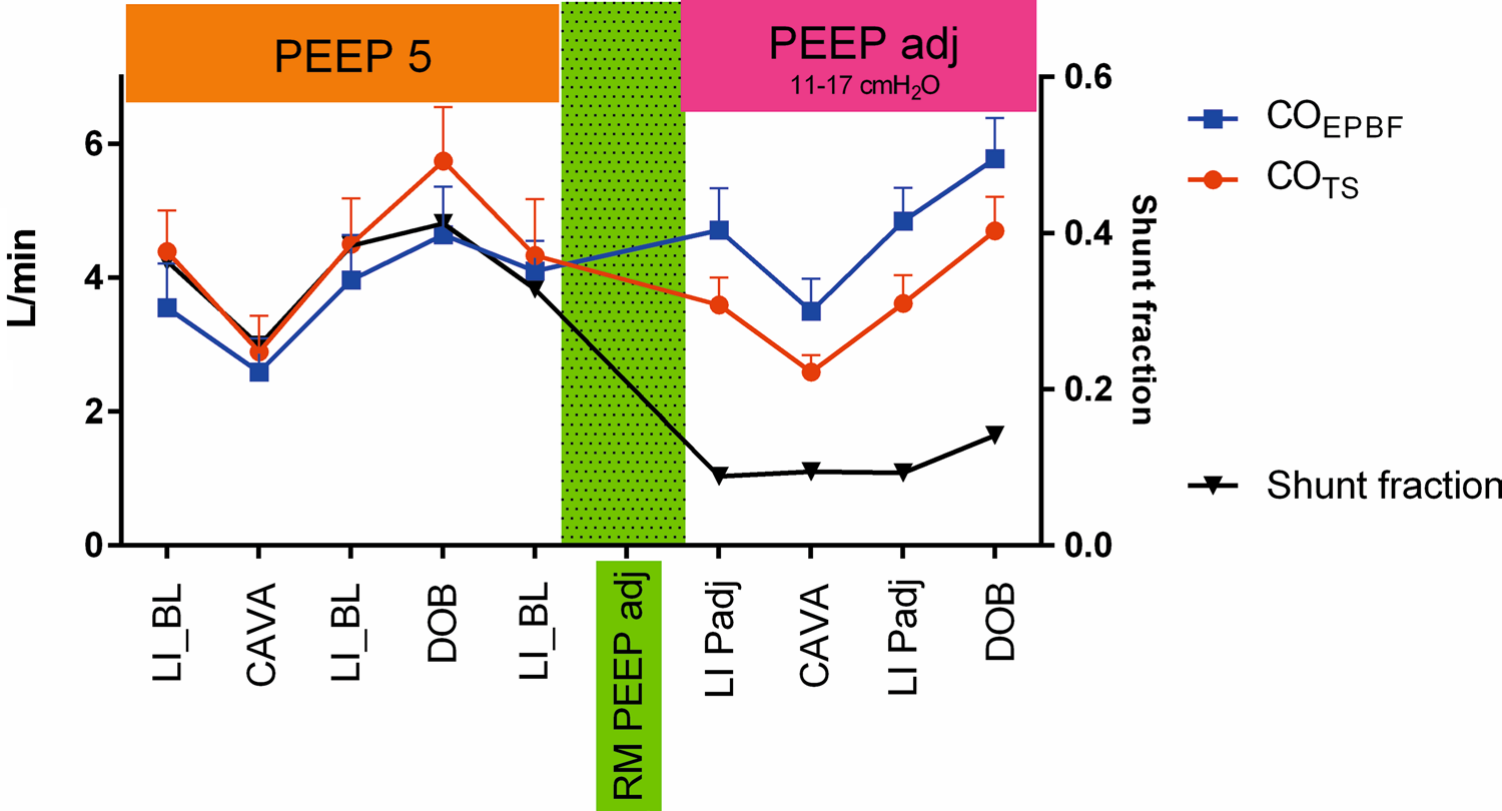
Timeline showing mean (SD) values for CO_EPBF_, CO_TS_ and shunt fraction throughout the lung injury (LI) protocol. *LI* lung injury, *CAVA* Preload reduction with balloon inflation in vena cava, *Dob* Dobutamine infusion, *RM* Recruitment manoeuvre, *Padj* PEEP adjustment

The calculated inherent precision of the CO_EPBF_ and CO_TS_ during initial baseline conditions were 9% and 6%, respectively. Mean CO during the lung injury protocol measured with CO_EPBFexp_ and CO_TS_ was 4.2 and 4.0 L/min and changed in average 30–35% (± 4–10%) during caval balloon inflation and dobutamine infusion (see Fig. 1 for event line). Data for CO measurements was normally distributed. No proportional bias was detected.

**Fig. 2:**
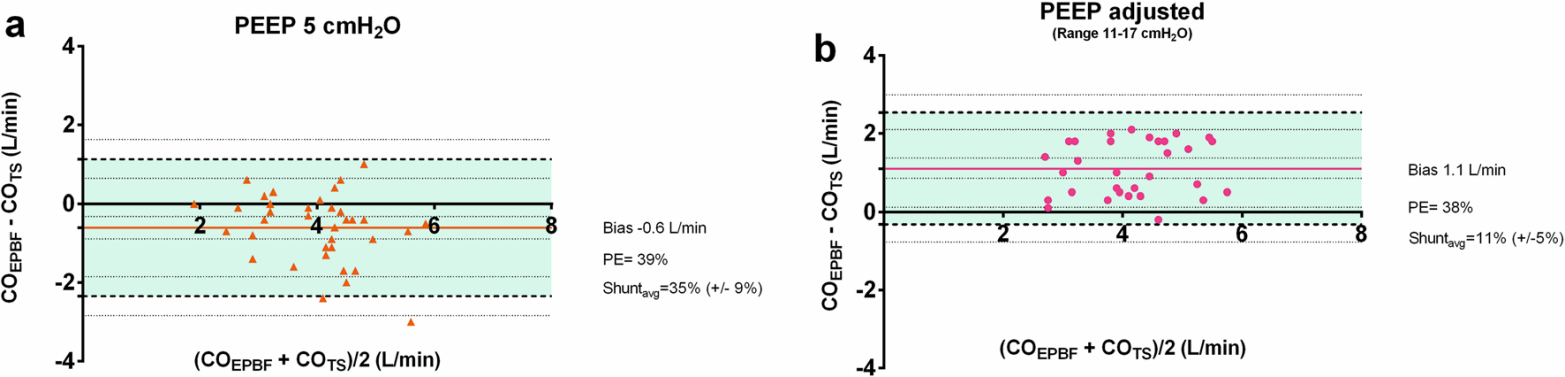
Bland–Altman plot showing **a** 37 paired values for CO_EPBF_ versus CO_TS_ during LI with high shunt fraction at PEEP 5 cmH_2_O (P5—orange triangles) and **b** 31 paired values after PEEP adjustment (Padj—pink dots). Bias is represented with a whole line with corresponding CI (dotted lines) and levels of agreement (LoA) are shown with broken lines with corresponding CI (dotted lines)

Bias (LoA) and PE for the CO_EPBF_ compared to CO_TS_ changed from baseline 0.5 (− 0.5 to 1.5) L/min and 30% to − 0.6 (− 2.3 to 1.1) L/min and 39% at LI_P5_ and finally 1.1 (− 0.3 to 2.5) L/min and 38% after recruitment manoeuvre and PEEP adjustment (see Fig. 2; Table 2).

**Table 2:** Mean cardiac output (L/min) and Bland–Altman results for CO_EPBF_ and CO_TS_ at different conditions and interventions with confidence intervals (CI) for bias and upper/lower level of agreement (LoA) and the percentage error (PE)

Condition	CO_EPBF_ (L/min)	CO_TS_ (L/min)	Bias (L/min)	CI_bias (L/min)	LoA (L/min)	CI_lower LoA (L/min)	CI_upper LoA (L/min)	PE (%)
HL_P5_	4.0	3.5	0.5	0.1 to 0.9	− 0.5 to 1.5	− 0.9 to − 0.2	1.2 to 1.9	30
LI_P5__BL	3.6	4.4	− 0.8	− 1.5 to − 0.2	− 2.7 to 1.0	− 3.3 to − 2.0	0.4 to 1.6	42
LI_P5__CAVA	2.6	2.9	− 0.3	− 0.6 to 0.1	− 1.5 to 0.9	− 2.0 to − 1.1	0.5 to 1.4	43
LI_P5__BL	4.0	4.5	− 0.5	− 1.2 to 0.1	− 2.3 to 1.2	− 2.9 to − 1.7	0.6 to 1.8	39
LI_P5__DOB	4.6	5.7	− 1.2	0.7 to 1.7	− 0.2 to 2.5	− 0.6 to 0.3	2.0 to 3.0	23
LI_P5__BL	4.1	4.3	− 0.2	− 0.8 to 0.3	− 1.8 to 1.3	− 2.3 to − 1.2	0.8 to 1.8	35
**LI**_**P5**_**_all**	**3.7**	**4.3**	**−** **0.6**	**−** **0.9** **to** **−** **0.3**	**−** **2.3** **to** **1.1**	**−** **2.6** **to** **−** **2.0**	**0.8** **to** **1.4**	**39**
LI_Padj__BL	4.7	3.6	1.1	0.6 to 1.6	− 0.3 to 2.6	− 0.9 to 0.2	2.1 to 3.1	41
LI_Padj__CAVA	3.5	2.5	1.0	0.6 to 1.5	− 0.3 to 2.3	− 0.7 to 0.2	1.9 to 2.8	52
LI_Padj__BL	4.8	3.6	1.2	0.8 to 1.7	− 0.1 to 2.5	− 0.5 to 0.4	2.1 to 3.0	36
LI_Padj__DOB	5.8	4.7	1.1	0.5 to 1.7	− 0.6 to 2.7	− 1.2 to − 0.0	2.2 to 3.3	35
**LI**_**Padj**_**_all**	**4.7**	**3.6**	**1.1**	**0.9 to 1.3**	**− 0.3 to 2.5**	**− 0.5 to − 0.0**	**2.3 to 2.7**	**38**

*LI*_*P5*_ lung injury at PEEP 5 cmH_2_O, *BL* baseline, *CAVA* preload reduction with balloon inflation in the vena cava, *DOB* dobutamine infusion, *LI*_*Padj*_ lung Injury after recruitment manoeuvre and PEEP adjustment

**Fig. 3:**
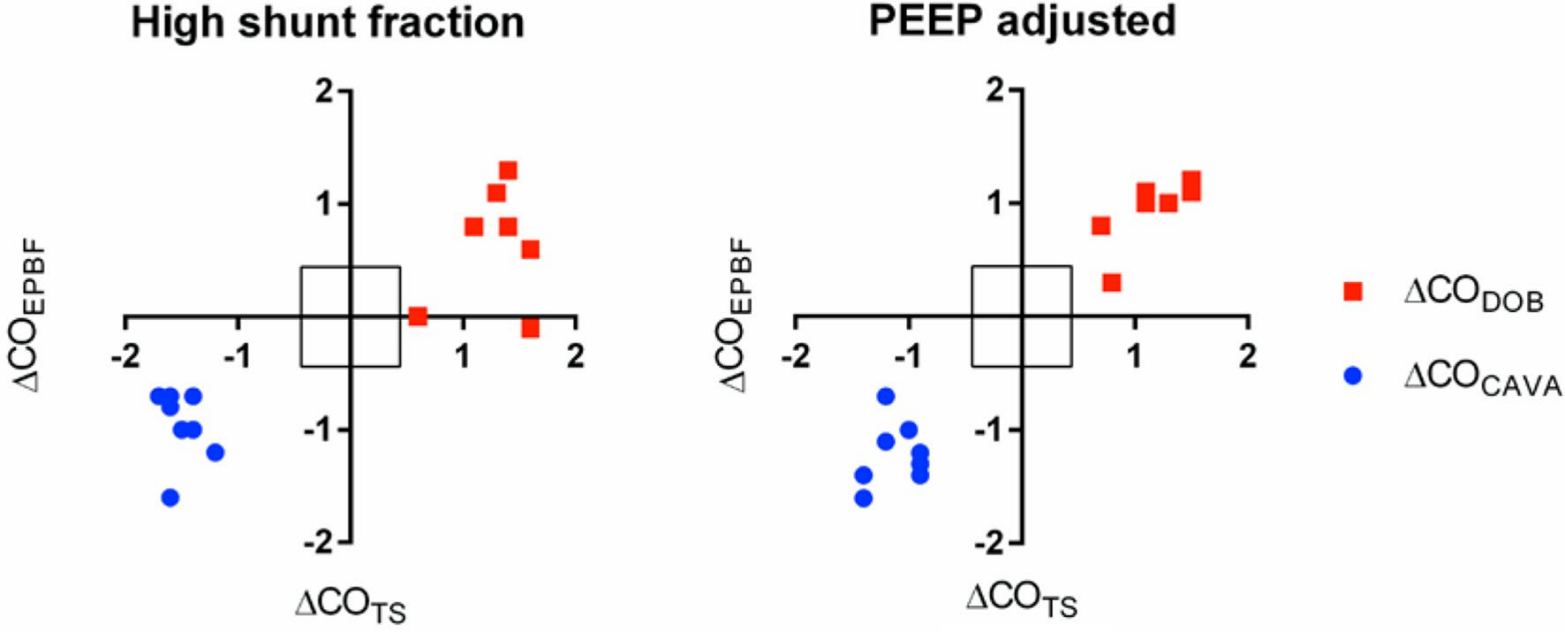
A four-quadrant plot showing total 30 paired delta values as measured with CO_EPBF_ and CO_TS_ during preload reduction with balloon inflation in vena cava (blue circles) and Dobutamine infusion (red squares) at high shunt fractions and after lung recruitment and PEEP adjustment

**Fig. 4:**
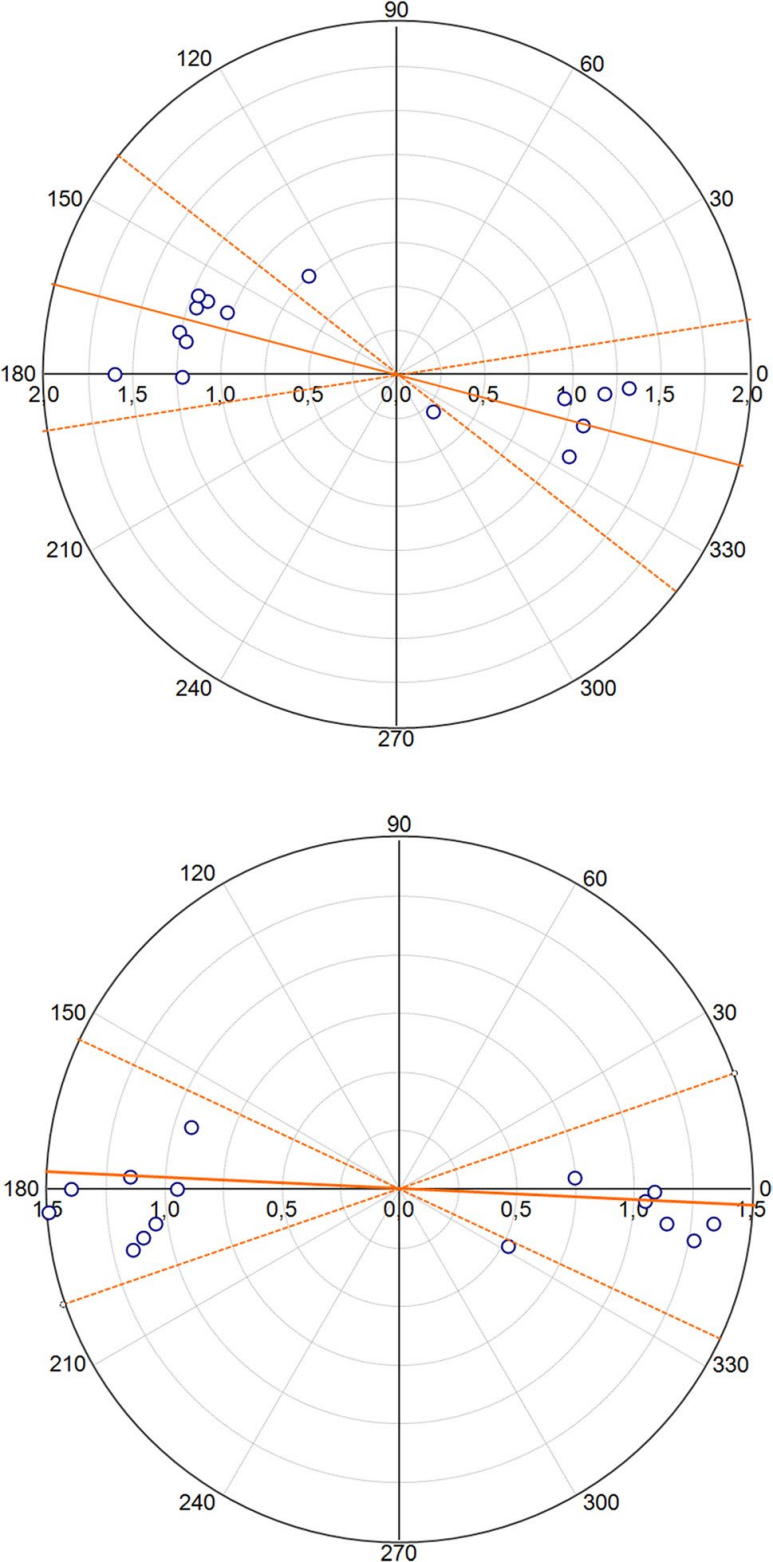
Polar plot for COEPBF with COTS as a reference during lung injury at high shunt fractions and after lung recruitment and adjustment. The radial length is the mean of the pairwise delta values of the reference method and the test method (L/min) and is shown with a whole orange line with corresponding CI (dotted lines). Data spread closely to the polar axis (whole black lines) indicate good trending

Concordance during LI_P5_ and LI_PAdj_, was 87 and 100% via the four-quadrant plot and 93 and 100% in the polar plot. The mean (95% CI) polar angle during LI_P5_ and LI_Padj_ was − 14.8° (− 40.0° to − 10.5°) and − 2.3° (−22.9° to 18.3°), respectively (see Figs. 3, 4).

## Discussion

We have evaluated the performance of the modified capnodynamic method based on expiratory holds in an experimental lung injury model resulting in high shunt fraction at PEEP 5 cmH_2_O and after lung recruitment with PEEP adjustment. In addition, both conditions were evaluated during major CO changes. CO_EPBFexp_, without any shunt correction, underestimated CO at high shunt fraction by 14% and overestimated CO by 30% after recruitment and PEEP adjustment. The ability to track changes was only marginally affected at high shunt and improved to 100% after lung recruitment as assessed by the four-quadrant and polar plot analyses.

Respiratory failure is a challenging situation for clinicians working in the operating theatre and intensive care unit. In severe cases, elevated shunt, hypoxemia and hypercapnia can lead to increased pulmonary vascular resistance and right ventricular failure, if not treated adequately. Mechanical ventilation is the mainstay of supportive treatment conveying oxygenation and ventilation at the lowest driving pressure possible. Mechanical ventilation providing continuous CO_EPBF_ monitoring has the potential to detect almost instantaneous hemodynamic changes, which can help the clinician to optimize oxygen delivery in real time via PEEP titration and adjustment of fluid and vasopressor therapy.

The capnodynamic method indirectly calculates the pulmonary blood flow based on CO_2_ elimination kinetics. CO_EPBF_ is equivalent to the non-shunted fraction of the CO and therefore potentially affected by disease states that increase the shunt fraction. The capnodynamic method based on inspiratory holds performed poorly in a porcine lung-lavage model. Precision was low during high shunt fraction and decreased further after PEEP elevation to 12 cmH_2_O, compared to the CO_TS_ [6]. Interestingly, even before the lavage, a paradoxical rise in CO_EPBFinsp_ was observed at PEEP 12 cmH_2_O [6]. The authors concluded that the observed increase in intrathoracic and airway pressure parallel to the inspiratory pause phases may have caused fluctuation in the pulmonary blood flow per se, possibly disturbing the carbon dioxide signal leading to errors in the obtained CO_EPBFinsp_ value [6]. The current improved breathing pattern, combining six normal breaths followed by three with added short expiratory hold lowers the average airway pressure during a set of nine breaths [5]. This probably decreases the variation in the pulmonary blood flow, providing more stable conditions for accurate calculations as observed when the CO_EPBFexp_ was challenged in a porcine model during various ventilatory and hemodynamic changes in healthy lungs [5].

In the current study CO_EPBFexp_ overestimated CO at higher PEEP levels without markedly affecting precision or trending ability. The expected increase in lung volume, as new regions were re-opened with the RM and kept open with the adjusted PEEP, was captured by the CO_2_-based estimation (ELV), an entity included in the capnodynamic equation (see Table 1). However, this entity has not been evaluated with the revised breathing pattern or during lung injury, per se. The reason for the overestimation at higher PEEP levels in the animal model is not entirely clear. At constant metabolism and ventilatory settings, any change in the elimination of CO_2_ can be explained by a combination of changes in (1) the effectiveness of the pulmonary blood flow, (2) the area for CO_2_ exchange, and the global ventilation–perfusion relationship of the lungs (V/Q ratio). These factors can be different between healthy and injured lungs [23]. The capnodynamic method is based on a homogenous lung model with regards to V/Q ratio. In the injured lung there will be a distribution of varying V/Q conditions over the lung, even after recruitment, especially over distended regions. How these affect the result of the prototype method is not fully understood, but could contribute to the observed over estimation at high PEEP in this study. Previous animal studies and our overall data show conflicting results in terms of higher PEEP (increased lung volume) and accuracy of the CO_EPBF_. Interestingly, in a recent clinical study (manuscript) where CO_EPBF_ was compared to transpulmonary thermodilution during open abdominal surgery, no over estimation was observed even at high PEEP levels (up to 20 cmH_2_O) [24].

The current experimental protocol using the improved capnodynamic method departs from our previous study [6] in three ways. Firstly, the animals had experienced caudal ischemia and reperfusion approximately 2 h before [7], possibly adding extra stress and inflammation; secondly, VILI was added to the conventional lavage to induce more severe respiratory failure and thirdly, an individualized recruitment manoeuvre with PEEP adjustment based on compliance, instead of arbitrarily raising it to 12 cmH_2_O. Despite the more extreme insult to the animals, percentage error for CO_EPBFexp_ was roughly unchanged (avg. 38 and 39%) during LI opposite to the high PE (avg. 70 and 75%) observed in the previous study with CO_EPBFinsp_. A finding consistent with the overall performance of CO_EPBFexp_ in healthy lungs.

The accuracy of CO_EPBF_ is dependent on the ventilation-perfusion status of the lung. When the shunt fraction is increased, CO_EPBF_ underestimates CO. This can be managed in two ways; either by incorporating shunt correction methods such as the iso-shunt diagram used by the NICO monitor [25] or by combining measurement of oxygen uptake and pulmonary blood flow (via the Fick equation) as applied in the capnotracking method developed by Peyton et al. [26], or alternatively by keeping the lung open with lung recruitment and PEEP adjustment. Recruitment manoeuvres are common in clinical practice both in the operation theatre and ICU and will most likely reduce any significant shunt fraction and improve the overall performance of the capnodynamic method in the commonly used PEEP range. Therefore, CO_EPBF_ could possibly be used as an independent physiological variable to adjust PEEP for best oxygen delivery as suggested by Gedeon et al., in a study of a small group of patients with acute respiratory failure [10]. For the clinician managing patients with respiratory failure in the operation theatre or ICU, perhaps the most important function of a hemodynamic monitor is to continuously and reliably detect relevant CO changes and response to treatment, as repeatedly shown by the capnodynamic method under experimental conditions.

Despite the severe lung injury, high shunt fraction, recruitment manoeuvre and PEEP adjustment the CO_EPBFexp_ had a PE of 38 and 39%, when compared to the highly accurate Transonic flow probe. Traditionally, a PE of 30% has been used as a priori to determine interchangeability between the studied and reference method [20]. However, it should be kept in mind that this cut off is primarily based on the inherent precision of the PAC calculating CO via thermodilution during stable hemodynamic situations and a simulation model [27, 28]. Based on the performance of the PAC, the 30% cut-off has recently been challenged by Peyton and Wong, where a PE of 45% was suggested when comparing new non-invasive CO methods in clinical situations when clinical benefit is anticipated [29].

The capnodynamic method calculates CO by changing the time between three out of nine breaths during controlled mechanical ventilation. Any triggered breath will affect the accuracy of CO_EPBF_ for the next nine breaths. However, the method includes an error function estimating the internal validation of the calculations (not used in this study). This function could theoretically be programmed to filter out CO_EPBF_ values with an elevated internal error, such as triggered breaths and therefore support the clinician with only stable calculations of pulmonary blood flow.

This study has several limitations. It is a small animal study where the induced respiratory failure is artificial, although the ischemia, reperfusion and VILI might have added to the clinical application. Each animal responded differently to lung injury and lung recruitment resulting in inter-individual differences in treatment and physiological status. As CO_EPBF_ calculations are based on human data and CO_EPBF_ and the reference method do not measure the same physiological variable, some difference is to be expected.

In this study, the performance of the revised capnodynamic method was evaluated during lung injury, with high shunt and after lung recruitment with PEEP adjustment. CO_EPBFexp_ showed good trending ability, especially when CO decreased. Accuracy was affected during high shunt and elevated PEEP although precision might be considered clinically acceptable. Clinical studies validating performance during high risk surgery and intensive care are underway.

## Electronic supplementary material

Below is the link to the electronic supplementary material.

Supplementary material 1 (DOCX 15 kb)
